# Multifunctional mussel-inspired copolymerized epigallocatechin gallate (EGCG)/arginine coating: the potential as an ad-layer for vascular materials

**DOI:** 10.1093/rb/rbw027

**Published:** 2016-07-13

**Authors:** Rifang Luo, Linlin Tang, Lingxia Xie, Jin Wang, Nan Huang, Yunbing Wang

**Affiliations:** 1 ^1^National Engineering Research Center for Biomaterials, Sichuan University, Chengdu 610064, China; 2 ^2^Key Lab of Advanced Technology of Materials of Education Ministry, Southwest Jiaotong University, Chengdu 610031, China

**Keywords:** Mussel chemistry, epigallocatechin gallate (EGCG), vascular devices, surface modification, multifunction

## Abstract

Surface properties are considered to be important factors in addressing proper functionalities. In this paper, a multifunctional mussel-inspired coating was prepared via the direct copolymerization of epigallocatechin gallate (EGCG) and arginine. The coating formation was confirmed by X-ray photoelectron spectroscopy and Fourier transform infrared spectra. The EGCG/arginine coating contained diverse functional groups like amines, phenols and carboxyls, whose densities were also tunable. Such mussel-inspired coating could also be applied as an ad-layer for its secondary reactivity, demonstrated by quartz crystal microbalance technique. Moreover, the tunable surface density of phenols showed potential ability in modulating endothelial cell and smooth muscle cell viability. The coatings rich in phenols presented excellent free radical scavenging property. Current results strongly indicated the potential of EGCG/arginine coatings to be applied as an ad-layer for vascular materials.

## Introduction

As generally accepted, cardiovascular diseases have the highest fatality rate in clinic and vascular stent has been demonstrated for its beneficial effects [1, 2]. Unfortunately, during angioplasty, the acute vessel wall injury often causes the increased incidence of restenosis, thrombosis and intimal hyperplasia [3–5]. Surface properties are considered to be important factors in addressing proper surface functionalities. Therefore, surface modification techniques are of significance in obtaining desired surface functionality. So far, the obtaining of proper biofunctions is still a challenge due to the insufficient reactive functional groups on the material surface. Thus, how to select a proper surface modification technique from the tool box and construct a multifunctional ad-layer is a hot topic in current research [6].

In relation to vascular materials, the complicated vascular microenvironment presents the basic requirements for obtaining desired surface functionalities like antithrombosis formation, antiproliferation and supporting rapid endothelialization [4, 7]. Construction of biocompatible ad-layers plays an important role in developing material/tissue interfaces with above multifunctions. With a careful scan of current published papers, an ad-layer for vascular material should not only cause no cytotoxicity but also possess reaction sites for further biomolecule functionalization. Inspired by mussel adhesive chemistry, a versatile polydopamine (PDA) coating was recently widely applied to modify various substrates, reported by Lee et al. [8]. Based on Schiff base or Michael addition reactions, PDA coating was famous to afford the immobilization of biomolecules like bone morphogenetic proteins, vascular endothelial growth factor, laminin and other peptides [9–11]. Moreover, PDA was also demonstrated to be biocompatible as an ad-layer [12]. Interestingly, our previous study had proved that after PDA formation, the retained catechols could affect the proliferation of smooth muscle cells (SMCs) which depends on the group densities [13]. This finding suggested the potential of PDA in modifying vascular stents, which also drove us to think what might happen on a phenol-rich surface to afford more evidence on this topic via investigating the effects on SMCs and ECs.

The co-existence of catechols and amines is demonstrated to be crucial to prepare robust mussel-based coating [14]. Based on this, a mussel-inspired coating formation procedure was established via the direct copolymerization between epigallocatechin gallate (EGCG) and arginine, which respectively played the roles as catechol and amine donors. As a matter of fact, a vascular stent is always suffering a microenvironment with severe oxidative stress, which is harmful to endothelial healing during the endothelial cell proliferation process. Our study here would like to address a possible protective coating for safe and affinitive endothelial growth. EGCG is a well-known green tea polyphenol and has excellent antioxidative activities, indicating the potential in vascular microenvironment [15]. Compared to PDA coating, with a stronger phenol group owner, EGCG/arginine coating might be phenol-rich and possess wider possibilities for effective surface modification. The multifunction were investigated in terms of secondary reactivity, selective performance on ECs/SMCs and free radical scavenging property of EGCG/arginine coating. To our knowledge, this is also the first investigation of using polyphenol/amine copolymerized ad-layers for modifying vascular materials with the function of modulating ECs/SMCs behavior and facing the oxidative stress in vascular environment. This job also aimed at enriching the study and application of mussel-inspired coatings.

## Materials and methods

### Materials

If not specially mentioned, reagents were local products of analytical grade. The micro-bicinchoninic acid (Micro-BCA) assay was obtained from Pierce Biotechnology Inc. (Rockford, USA). The EGCG, arginine, bivalirudin (BVLD) are bought from Sigma-Aldrich. Various reagents utilized in this work for the hemocompatibility and cytocompatibility analysis were provided from the professional manufacturers (details were mentioned in the experimental part).

### Preparation of EGCG/arginine copolymerized coating

**Scheme 1.**
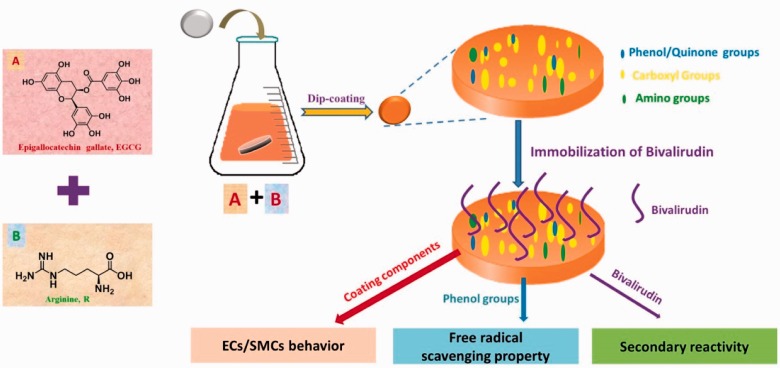
The brief work of this paper on the preparation of EGCG/arginine coating and the investigation of the multifunction.

EGCG/arginine coatings were fabricated on mirror polished 316 L stainless steel (SS) (Φ = 10 mm) at room temperature in the mixture solution of EGCG and arginine, dissolved in Tris buffer solution (pH 8.5) at diverse input concentrations and reacted for 24 h. After coating formation, they were then ultrasonically cleaned with deionized water and named as EGCG/R-*x*/*y*, where R is the abbreviation of arginine, *x* and *y* represented the final concentration of EGCG and arginine (mg/ml), respectively. The concentration of EGCG and L-Arg was shown respectively in Table 1 and the schematic was shown in Scheme 1. For some special test, coatings were deposited on Au-coated single crystal quartz (for quartz crystal microbalance (QCM) test) because of the inherent adhesive ability of catechols on various substrates.

**Table 1:** The parameters and labels for diverse EGCG/arginine coatings

Samples	EGCG/R-4/2	EGCG/R-2/2	EGCG/R-2/4
EGCG (mg/ml)	4	2	2
Arginine (R) (mg/ml)	2	2	4

### Surface characterization

The attenuated total reflectance Fourier transform infrared spectroscopy (ATR-FTIR, ranging from 4000–500 cm^−1^, NICOLET 5700) was used to test the chemical structure of the EGCG/arginine coating. Moreover, X-ray photoelectron spectroscopy (XPS, Perkin-Elmer 16PC) was utilized to characterize the surface chemical compositions, with a monochromatic Al Kα excitation radiation (1486.6 eV). A containment carbon (C1s = 284.7 eV) was used to calculate the binding energies. The high-resolution information was obtained via peak fitting using Xpspeak 4.1. The surface morphology test was done by Atomic Force Microscope (CSPM5000) and the water contact angles (WCA) was measured using DSA100 (Krüss, Hamburg, Germany).

An acid orange II (AOII) method was adopted for the amine group content quantification as described before [16]. Carboxyl group density quantification was determined via toluidine blue O method [17]. Because phenol groups could lead to the reduction of Cu^2+^ to Cu^+^, a modified Micro-BCA method was adopted to quantitate phenol group content according to the formation of BCA/Cu^+^ complex [18].

### Secondary reactivity evaluation

QCM (Q-Sense AB, Sweden Company) measurement is a facile tool to investigate the potential of ad-layer for biomolecule immobilization [18]. Before the test, each EGCG/arginine coating was coated on the Au-coated single crystal quartz (Φ = 10 mm). Each crystal was initially exposed to 20 mm PBS (pH = 7.4) fluid at a flow rate of 50 μl/min, in order to remove the unstable surface coating components. After that, BVLD, known as a direct thrombin inhibitor, was then passed through the chamber in contact with the crystal to test the secondary reactivity [19]. The Sauerbrey relation indicated the relationship between the frequency shift (Δf) and the adsorbed mass (Δ*m*) [20]:

(1) Δm=Δf×Cn

where *n* (*n* = 1, 3, 5, …) was the overtone number and *C* (*C* = 17.7 ng/cm^2^ HZ^−1^ at *fn* = 5 MHz) was the mass-sensitivity constant. Moreover, normal biomolecule immobilization was done on EGCG/arginine coated 316 L SS samples and the subsequent effects on anti-platelet adhesion were investigated.

### Endothelial cell attachment and proliferation

Based on Jaffe *et al*., human umbilical vein endothelial cells were isolated from newborn umbilical cord [21]. Following isolation, cells in passage 2 were used. ECs were seeded at a concentration of 5 × 10^4^ cells/per sample to investigate the cell proliferation behavior, cultured using M199 media (Gibco, USA) supplemented with 15% FBS (Sigma, USA) in humidified air containing 5% CO_2_ at 37°C. Samples were taken out at the predetermined time points (2 h, 1 day and 3 day). After that, samples were washed with PBS and cells were fixed using 2.5% glutaraldehyde for 4 h. Cells were then stained with rhodamine-phalloidin (lucifuge for 20 min). Fluorescent microscopy (Zeiss, Germany) and IPWin60C (software) was used to observe adhered cells and evaluate cell adhesion and proliferation results.

### Protective effect of ECs against H_2_O_2_ injury

The endothelium could maintain the balance between vasodilation and vasoconstriction. Once broken, endothelial dysfunction occurs and causes the damage to the arterial wall, followed with the SMC proliferation and migration, thrombogenesis and fibrinolysis [22]. Usually, vascular implants are surrounded with oxidative stress injury, which sometimes is ignored when design coatings or ad-layers of vascular materials. Herein, we evaluated the ability of EGCG/arginine ad-layers for free radical scavenging property via 2,2-Diphenylpicrylhydrazyl (DPPH) assay. In brief, freshly prepared samples were placed in a 24-well plate, and 200 μl of DPPH solution (0.1 mm in 95% ethanol) was added on each sample surface and reacted for another 30 min in dark, following a microreader in 517 nm. Moreover, the protective effects of ad-layers on ECs were also investigated. ECs were cultured on samples for 24 h and then half of the samples were transferred to a new plate, followed by the addition of H_2_O_2_ (final concentration was 2 mm) to create cell injury and cultured for another 3 h. After that, cells were fixed and visualized by ﬂuorescent microscopy.

### Smooth muscle cell proliferation and apoptosis

SMCs were isolated from the tunica media of newborn umbilical cord [23]. SMCs between passages 5 and 9 were used. To investigate the SMC proliferation cultured on different EGCG/arginine coating surface, cells were seeded at a concentration of 2 × 10^4^ cells/per sample at the predetermined time of 1, 3 and 5 day, incubated in 1 ml of Dulbecco's Modified Eagle Medium-F12 culture media supplemented with 10% FBS at 37°C. For cell apoptosis evaluation, SMCs were seeded at a density of 2 × 10^4^ cells/per sample for 1 and 3 day. After that, a 1:1 mixture of acridine orange (100 mg/ml) and propidium iodide (100 mg/ml) solution were freshly prepared to stain the adhered cells at 37°C for 5 min, and then immediately viewed in a ﬂuorescence microscope.

### Statistical analysis

For statistical analysis, at least five samples for each experimental condition or tested time points were used. The quantitative results were reported as mean ± standard deviation (SD) and the one-way analysis of variance was adopted for the statistical analysis. Moreover, the statistical significance was recorded at the *P* values when less than or equal to 0.05 (*P ≤ 0.05, **P ≤ 0.01, ***P ≤ 0.001).

## Results and discussion

### Surface properties

**Figure 1.**
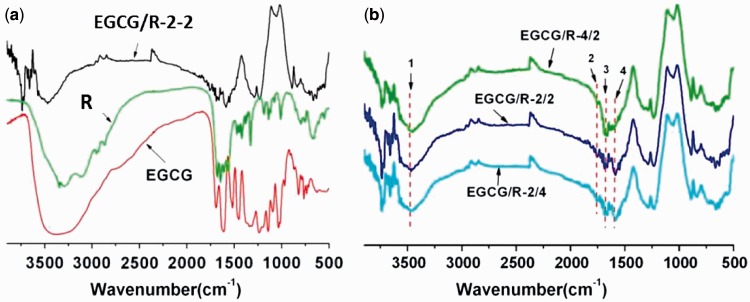
(A) ATR-FTIR results of arginine, EGCG and EGCG/R-2/2 coating and **(B)** the comparison of different EGCG/arginine coatings

Our previous study showed that catechols and amines together in an alkali solution would induce benzene ring coupling, Michael addition and Schiff base reactions [24]. ATR-FTIR spectroscopy was used to analysis the chemical structures of the modified surfaces. As shown in Fig. 1a, after the copolymerization of EGCG and arginine, the coating surface retained the functional groups of donor materials. The wide peak (peak 1) around 3200–3500 cm^−1^ indicated the polar components, which would possibly be an amine, hydroxyl and carboxyl groups. Seen in Fig. 1b, peaks at 1545= cm^−1^ (peak 4, N–H scissoring vibrations), 1650 cm^−1^ (peak 3, C N resonance vibrations in aromatic ring), 1758 cm^−1^ (peak 2, O-C = O vibrations), 2920 and 2850 cm^−1^ (aliphatic C–H stretching vibrations) were clearly observed. These results demonstrated the successful coating formation and functional groups retention. Interestingly, the relative concentration of input donor materials would cause different peak intensity on as-deposited coatings. Peak 3 of EGCG/R-4/2 had a strong intensity compared to peak 4, while this was opposite in EGCG/R-2/2 and EGCG/R-/2/4, who had a relatively higher input concentration of amine donors.

**Figure 2.**
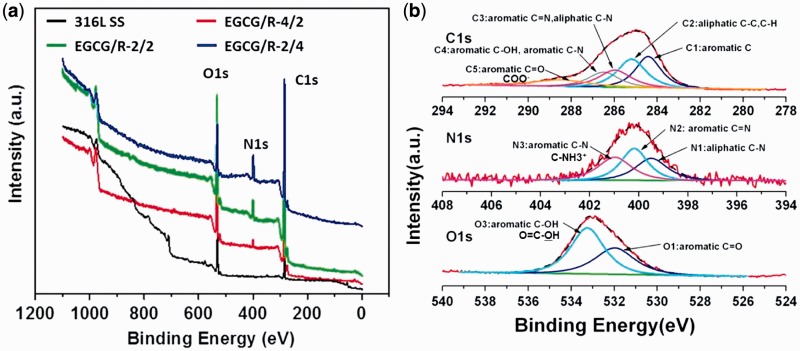
(A) XPS wide scan of different sample surfaces and **(B)** high-resolution survey of EGCG/R-4/2 coatings on C1s, N1s and O1s

For the detailed scan of chemical components, XPS data were shown in Fig. 2a and b. The appearance of nitrogen indicated the successful deposition of EGCG/arginine ad-layers and the relative intensity of nitrogen increased with the increased input concentration of arginine. Taking EGCG/R-4/2 as an example, the high-resolution survey of C1s, N1s and O1s was shown in Fig. 2b. The curve fitted results effectively demonstrated the retention of functional groups after coating formation (phenols, carboxyls and amines). The element ratio of different EGCG/arginine coatings were shown in Supplementary Table S1 (Supplementary Material). The retention of oxygen and nitrogen was associated with the input concentration of donor materials.

**Figure 3.**
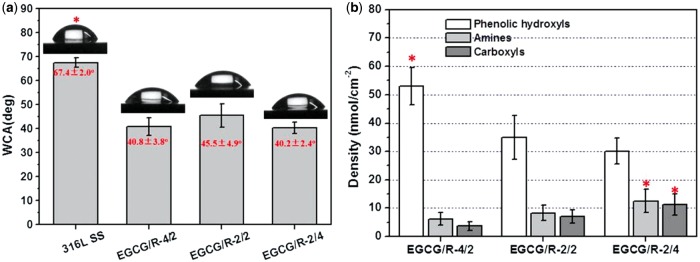
(A) WCA results and **(B)** quantitative determination of diverse functional groups. Data expressed as mean ± SD (*P < 0.05)

The results of WCA (Fig. 3a) and quantitative determination of surface functional groups (Fig. 3b) were also presented. With a good agreement with FTIR and XPS results, the polar components retention on EGCG/arginine coatings caused an increased hydrophilicity compared with 316 L SS. Moreover, such coatings are capable to afford diverse functional groups, which is useful for further biomolecule or diverse biomolecules functionalization based on unique immobilization chemistry. There are three typical functional groups (phenols, amines and carboxyls) existed after EGCG/arginine coating formation. These results also implied that the species and densities of functional groups could also be tunable via proper donor materials (amines and phenols) selection and regulation of the polymerization conditions. The morphologies of diverse coatings were shown in Supplementary Fig. S1 (Supplementary Material). Because, such polymerization process is PDA like more input EGCG concentration would cause faster copolymerization rate and could induce higher roughness.

### Secondary reactivity evaluation

**Figure 4.**
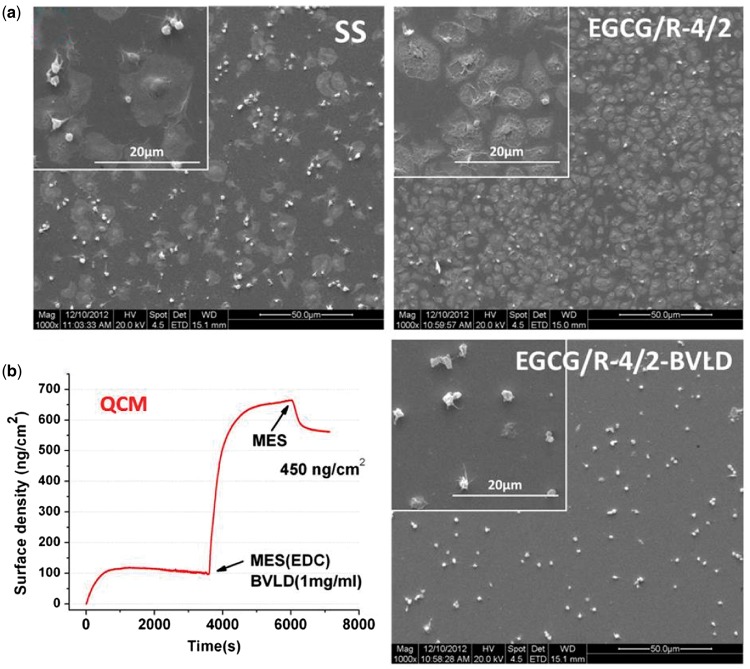
(A) Morphology of platelet adhesion and activation on different surfaces and **(B)** QCM monitoring of bivalirudin (BVLD) immobilization onto EGCG/R-4/2 coating surface

PDA is well-known due to its versatile function for coating various materials and performed as a star ad-layer for biomolecule immobilization to achieve further biofunctionality. As a PDA-like ad-layer, the secondary reactivity of EGCG/arginine coating also deserves attention. Herein, the EGCG/R-4/2 coating was selected to test its ability for immobilizing biomolecules. BVLD, a direct thrombin inhibitor [19], was adopted as the tested biomolecule and the corresponding result on platelet adhesion was also investigated. Fig. 4a showed that EGCG/R-4/2 coating could cause severe platelet adhesion and activation compared with 316 L SS. However, after BVLD immobilization, the amount of adhered and activated platelets on EGCG/R-4/2-BVLD significantly decreased, indicating the high activity of immobilized biomolecule. Fig. 4b showed that the amount of covalently immobilized BVLD reached up to 450 ng/cm^2^. These results have provided evidence for the secondary reactivity of EGCG/arginine ad-layer based on ( N-(3-dimethylaminopropyl)-N'-ethylcarbodiimide) EDC method (reactions between amines and carboxyls). It is also believed that phenol/quinone groups on EGCG/arginine coating could also be utilized to binding biomolecules (data not shown), just like PDA coating.

### Attachment and proliferation of endothelial cells (ECs)

**Figure 5.**
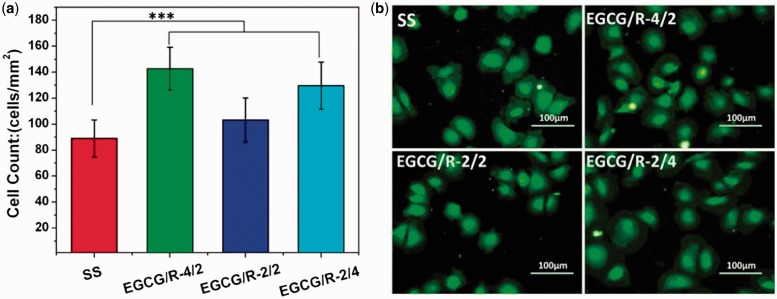
(A) Cell counting results of adhered endothelial cells and **(B)** cytoskeletal actin stains of adhered endothelial cells cultured for 2 h. Data expressed as mean ± SD (***P < 0.001)

It is of great importance to understand how ECs interact with implanted materials and the rapid survival of implants is often associated with the re-endothelialization process [25]. PDA has already been proved as an ad-layer with good ECs affinity, which might be ascribed to the maintaining of natural properties of serum proteins after interaction with PDA surface components (catechols and amines) [26]. What is the performance of EGCG/arginine coating on endothelial cell growth is also interesting. Figure 5 presented the endothelial cell counting and staining results after cultured for 2 h. Overall, the amount of adhered cells on EGCG/arginine coatings was larger than those on SS surface (obvious larger on EGCG/R-4/2 and EGCG/R-2/4 surfaces). EGCG/arginine coatings possessed the similar functional groups as PDA coating, which might be a possible reason for serum protein adsorption and could perform as a good cell adhesion site.

**Figure 6.**
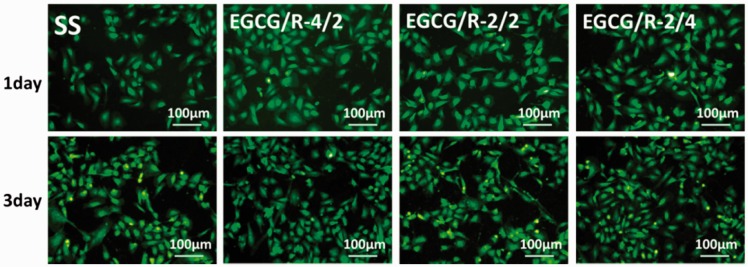
Endothelial cell staining results after the proliferation for 1 and 3 day cultured on different samples

Generally accepted, good cell adhesion property plays an important role in cell survival. Cell adhesion ability often causes the subsequent proliferation behavior. As shown in cell staining results (Fig. 6), except EGCG/R-2/2 coating, other EGCG/arginine coating surface showed an equivalent or even better affinity for endothelial cell proliferation, compared with 316 L SS. The cell viability results were shown in Supplementary Fig. S2 (Supplementary Material), which also indicated the similar phenomenon as shown in cell staining results. Surface components like phenols are in favor of serum protein adsorption and could support nice cell proliferation environment. However, there are three main functional groups retained on sample surfaces, and the complex effect on cell adhesion and proliferation has not ever been investigated. More factors like surface charge might also be a reason (to be done in the next step). Nevertheless, the tunable preparation of EGCG/arginine coating makes it possible to prepare the compatible surface for endothelial cells.

### Protective effect against H_2_O_2_ injury

Under the environment of atherosclerosis, lesions are in a state of oxidative stress injury. A large amount of reactive oxygen species (ROS) is harmful and could induce endothelial cell apoptosis and cause the increased synthetic phenotype of SMC [27]. How can the materials help to protect cells against free radical injury is also important that needed to be considered. Thus, the free radical scavenging ability of EGCG/arginine coatings were tested in this study, using DPPH assay.

**Figure 7.**
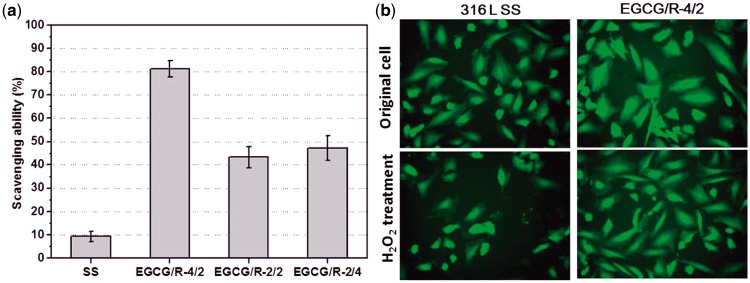
(A) The free radical scavenging ability of different samples and **(B)** the protective effect of ECs against H_2_O_2_ injury with EGCG/R-4/2 and 316 L SS samples

As shown in Fig. 7a, EGCG/R-4/2 coating which had the highest retention of phenol groups presented the best free radical scavenging results (near 80%), compared with SS and other EGCG/arginine coatings. EGCG is an excellent antioxidant and it is easy to be understood that, more retention of EGCG functional groups (phenol groups) are in favor to make better free radical scavenging ability. Within this finding, we specially investigated the coating ability of EGCG/R-4/2 on the protective effect of ECs against H_2_O_2_ injury, which could mimic the ROS injury to cells. It is obvious that, after H_2_O_2_ injury and cultured for another 3 h, the shape of ECs cultured on SS samples were destroyed and no more endothelial-like (Fig. 7b). Interestingly, after H_2_O_2_ treatment, cells cultured on EGCG/R-4/2 coating surface still appeared as the ‘paving stone’ like shape, which is a normal and functional endothelial phenotype. The most possible and powerful factor to direct ECs rate after H_2_O_2_ injury should be the difference on cultured substrates, where ECs could be protected against ROS injury by EGCG/R-4/2 coating, due to its excellent free radical scavenging property. A protective and multifunctional coating substrate could not only support cell adhesion and proliferation but also play the role as an envoy to help cells to survival in the complex microenvironment.

### Proliferation and apoptosis of smooth muscle cells

It is generally accepted that the proliferation of SMCs still remains a challenging clinical problem after stent implantation and is principally responsible for in-stent restenosis [4]. Several studies have shown that polyphenols like EGCG and gallic acid could not only inhibit SMCs adhesion and migration but also to a certain extent, could induce the apoptosis of SMCs via different approaches. There are some reported possible factors associated, including down-modulating nuclear factor-κB expression, affecting SMC’s integrin β1 expression, inhibiting the activation of pro-matrix metalloproteinase-2 and affecting the binding to extracellular matrix proteins [28–31]. Although the reports are focusing on drug effect, the dose of polyphenols is associated with the inhibitory effects. So can this inhibit the effect of phenols be reserved after phenol-containing coating formation?

**Figure 8.**
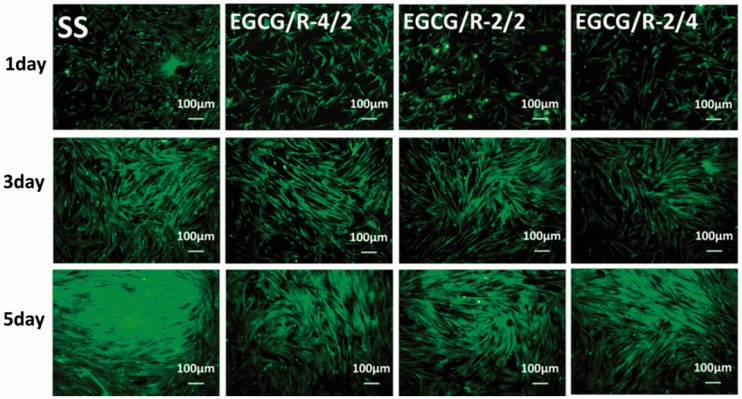
Smooth muscle cells staining on different surfaces after culturing for 1, 3 and 5 days

Figure 8 represented the proliferation results of SMCs on different sample surfaces. After 1 day culture, SMCs were more isolated on the EGCG/arginine coating surfaces compared to the 316 L SS surface. Moreover, during culture, SMCs on 316 L SS presented a rapid proliferation rate. After 5 days proliferation, compared with 316 L SS, all the EGCG/arginine coatings showed a significant lower adhered cell coverage rate of SMCs, which strongly indicated that EGCG/arginine coating, due to the retaining of high phenol groups, could significantly inhibit the proliferation of SMCs.

**Figure 9.**
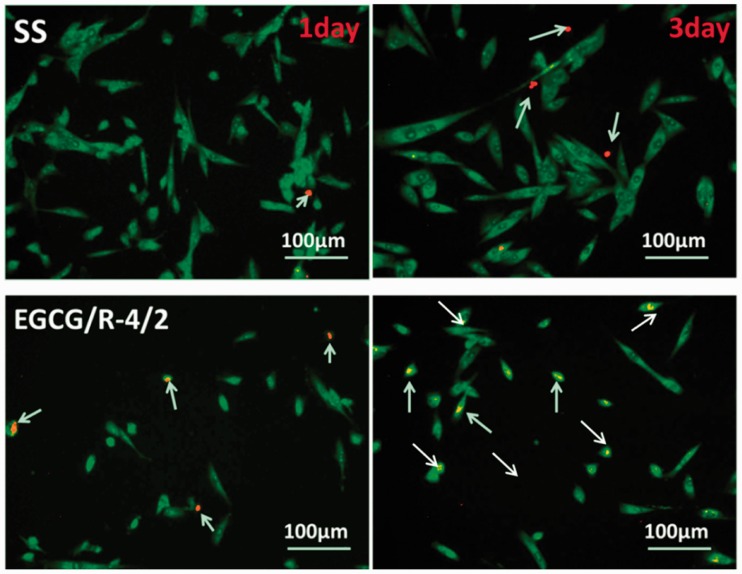
Apoptosis of SMCs cultured on 316 L SS EGCG/R-4/2 coating surface after 1 and 3 days culture

Polyphenols could induce SMCs apoptosis to some extent. In this job, we also investigated the apoptosis of SMCs on the phenol-rich EGCG/R-4/2 coating surface. As shown in Fig. 9, there was a significantly higher degree of green fluorescence but lower degree of red/orange fluorescence of SMCs cultured on 316 L SS surface, indicating the good affinity of 316 L SS to SMCs. The SMCs cultured on EGCG/R-4/2 coating surface were more isolated and presented a ‘rod-like’ shape. Moreover, a higher degree of red/orange fluorescence cells was observed, proving that phenol-rich coating could induce SMC apoptosis and thus was not a suitable substrate for SMCs growth.

An ad-layer, which does no harm to ECs proliferation and meanwhile could inhibit SMCs proliferation sound charming for vascular implants. Typically in this job, as a phenol containing coating, the phenol groups induced cell fate of ECs and SMCs deserved attention. The cell proliferation of ECs and SMCs affected by EGCG drug under different concentrations were also investigated. According to Supplementary Figs S3 and S4 (Supplementary Material), SMCs were more sensitive to EGCG concentration and shown cytotoxicity at a concentration of 100 μm. The toxicity effect of ECs was observed when the EGCG concentration reached up to 200 μm. This result also helped to evidence the effect of EGCG/arginine coatings on supporting EC proliferation and inhibiting SMC proliferation. Overall, based on the main topic of this job, a mussel-inspired copolymerized EGCG/arginine coating is multifunctional in terms of secondary reactivity, directing ECs and SMCs behavior and excellent free radical scavenging property. Current data strongly indicated the potential of EGCG/arginine coating as an ad-layer for vascular materials.

## Conclusion

This study demonstrated the successful coating formation based on the mussel-inspired chemistry. The copolymerized EGCG and arginine coating could possess diverse functional groups like amines, phenols and carboxyls, whose densities were also tunable. The coatings provided secondary reactivity for further biomolecule immobilization and presented excellent free radical scavenging ability. Moreover, the effect of EGCG/arginine coating in directing EC and SMC behavior make itself a potential ad-layer for vascular materials, especially when applied to modify vascular stents or grafts.

## Supplementary data

Supplementary data is available at REGBIO online.

Supplementary Table S1
