# Supplementary material for: Multifunctional mussel-inspired copolymerized epigallocatechin gallate (EGCG)/arginine coating: the potential as an ad-layer for vascular materials
Source: Regen Biomater. 2016 Jul 13;3(4):247–55. doi: 10.1093/rb/rbw027 (PMC5987673; doi:10.1093/rb/rbw027)
Supplement: Supplementary Table S1 [file Supplymentary_Material.docx]

Supplementary Material

Multifunctional Mussel-Inspired Co-Polymerizaed Epigallocatechin Gallate (EGCG)/Arginine Coating: the Potential as an Ad-layer for Vascular Materials

Rifang Luo^1,2, ▏^, Linlin Tang^2, ▏^, Lingxia Xie^2^, Jin Wang^2*^, Nan Huang^2^, Yunbing Wang^1,2*^

^1^ National Engineering Research Center for Biomaterials, Sichuan University, Chengdu 610064, China

^2^ Key Lab of Advanced Technology of Materials of Education Ministry，Southwest Jiaotong University，Chengdu 610031，China

*Correspondence address: National Engineering Research Center for Biomaterials, Sichuan University, Chengdu 610064, China. Tel：+86 28 85415280; E-mail: [yunbing.wang@scu.edu.cn](mailto:yunbing.wang@scu.edu.cn) (Yunbing Wang); and Key Lab of Advanced Technology of Materials of Education Ministry, Southwest Jiaotong University, Chengdu 610031, China. Tel: +86 28 87634148; E-mail: [jinxxwang@263.net](mailto:jinxxwang@263.net) (Jin Wang).

^▏^The first two authors contributed equally to this work.

**Content**:

Table S1 (element ratio of XPS results)

Figure S1 (AFM data)

Figure S2 (Endothelial cell viability test using cck-8 assay)

Figure S3 (Endothelial cell proliferation)

Figure S4 (Smooth muscle cell proliferation)

Table S1

Table S1. Elemental ratio of different EGCG/arginine ad-layers.

| Sample | C(%) | N(%) | O(%) |
| --- | --- | --- | --- |
| EGCG/R-4/2 | 70.7 | 4.0 | 25.3 |
| EGCG/R-2/2 | 70.5 | 5.1 | 24.4 |
| EGCG/R-2/4 | 69.1 | 7.4 | 23.4 |

Figure S1


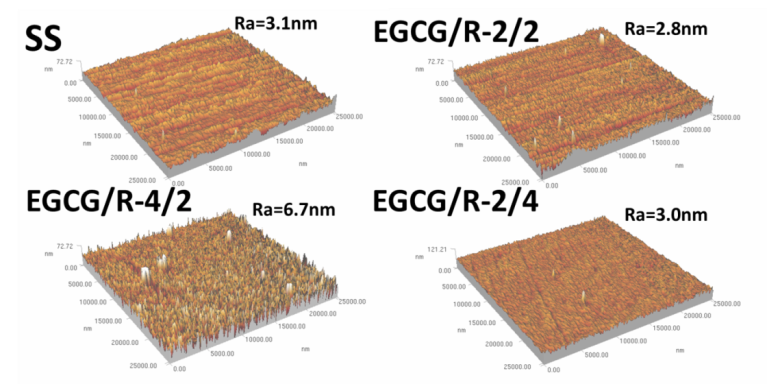


Figure S1. AFM results of different surface (coatings are prepared on 316 L SS surface).

Figure S2.


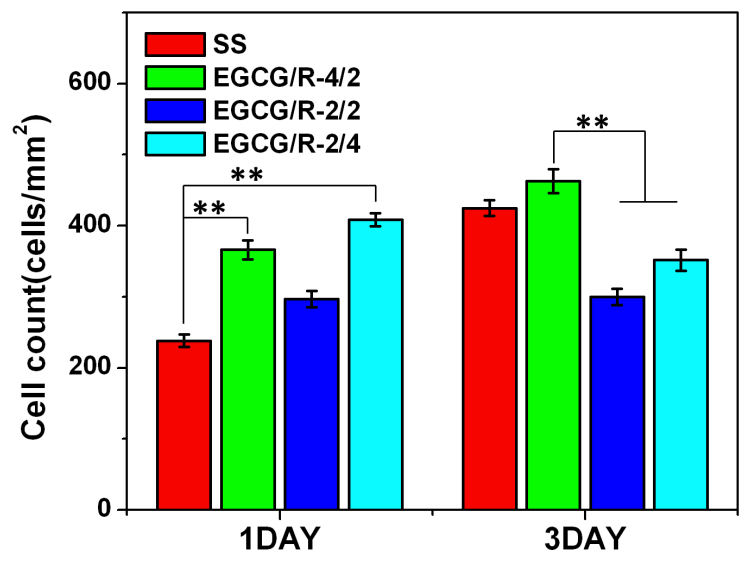


Figure S2. Endothelial cell viability after cultured for 1 day and 3 days.

Figure S3


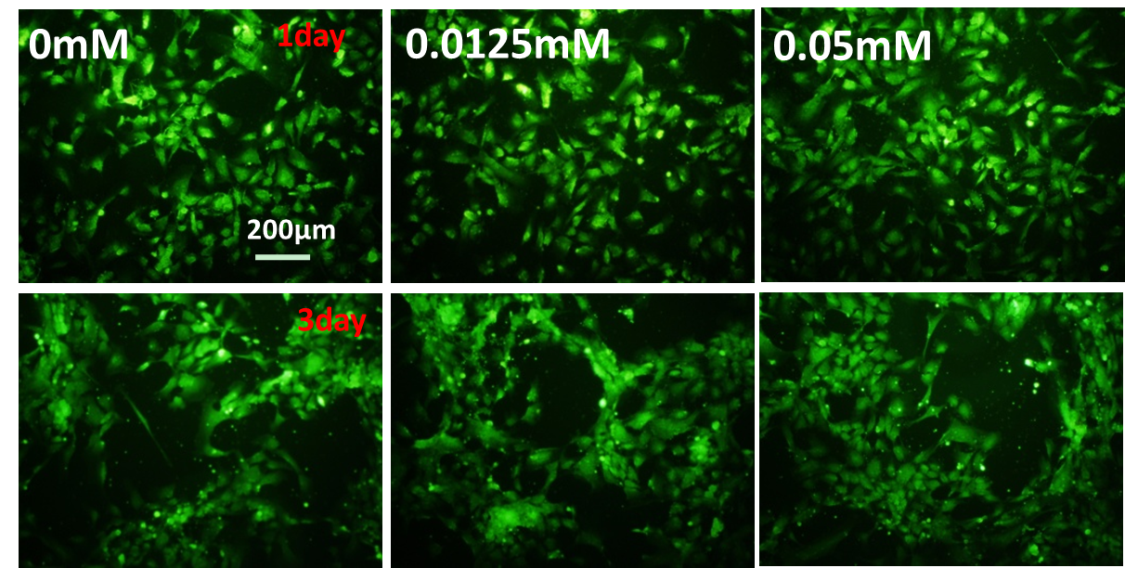


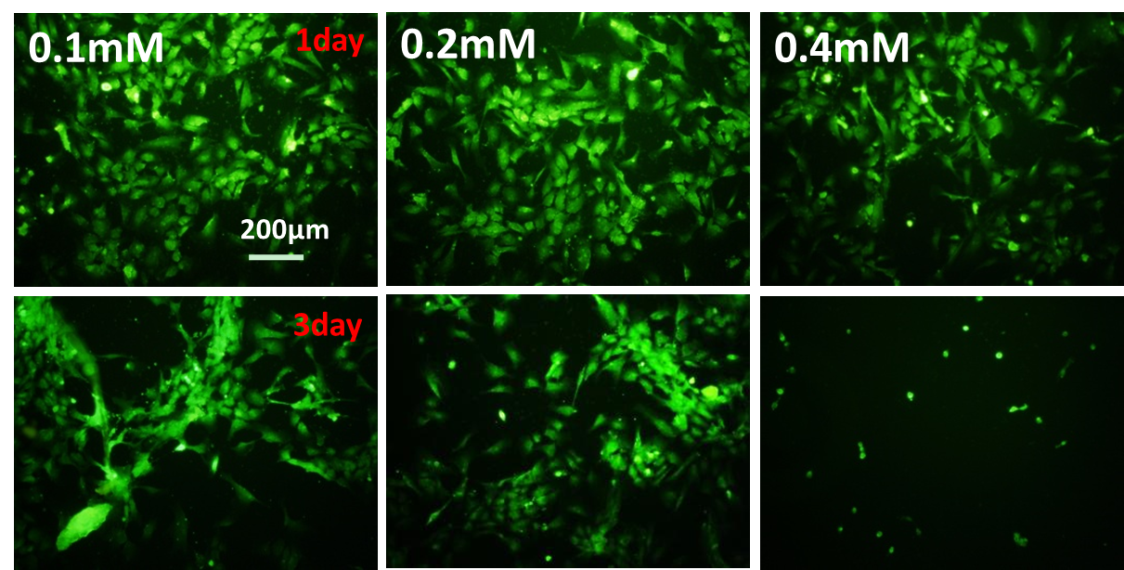


Figure S3. Endothelial cells cultured on plates incorporated with different EGCG concentrations.

Figure S4


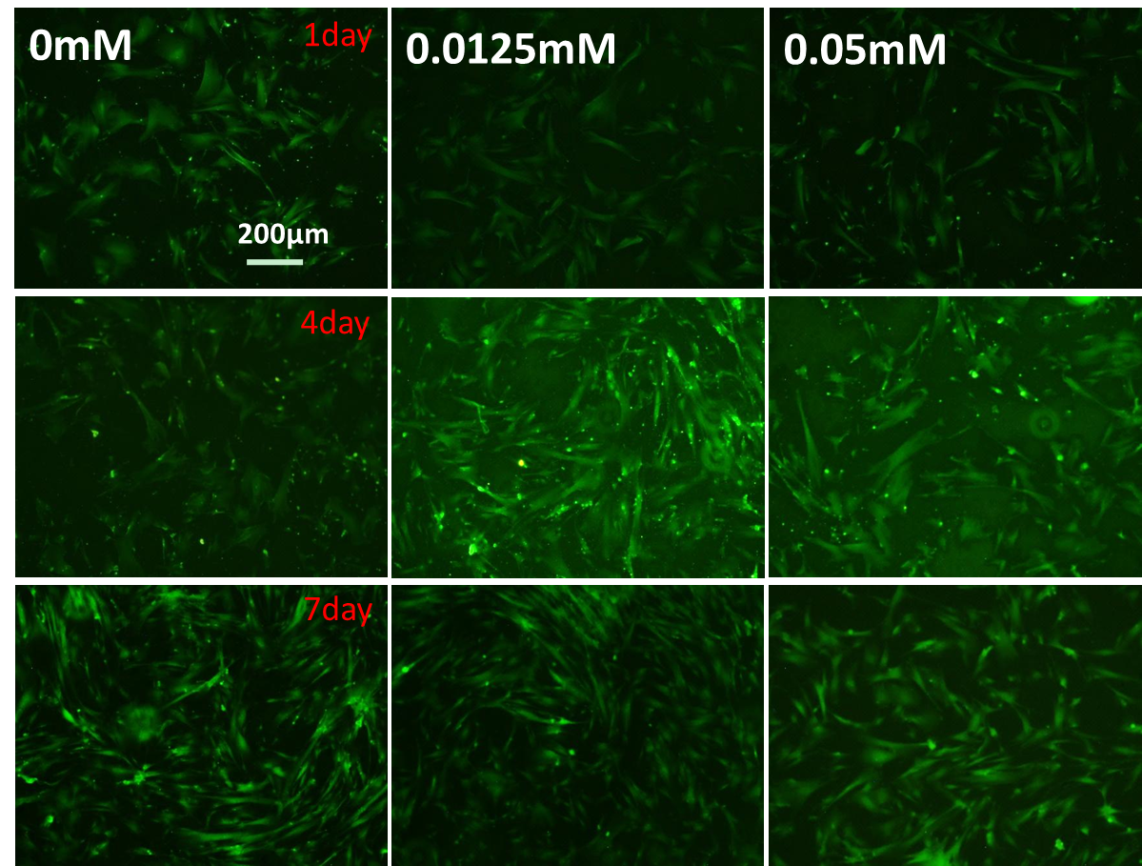


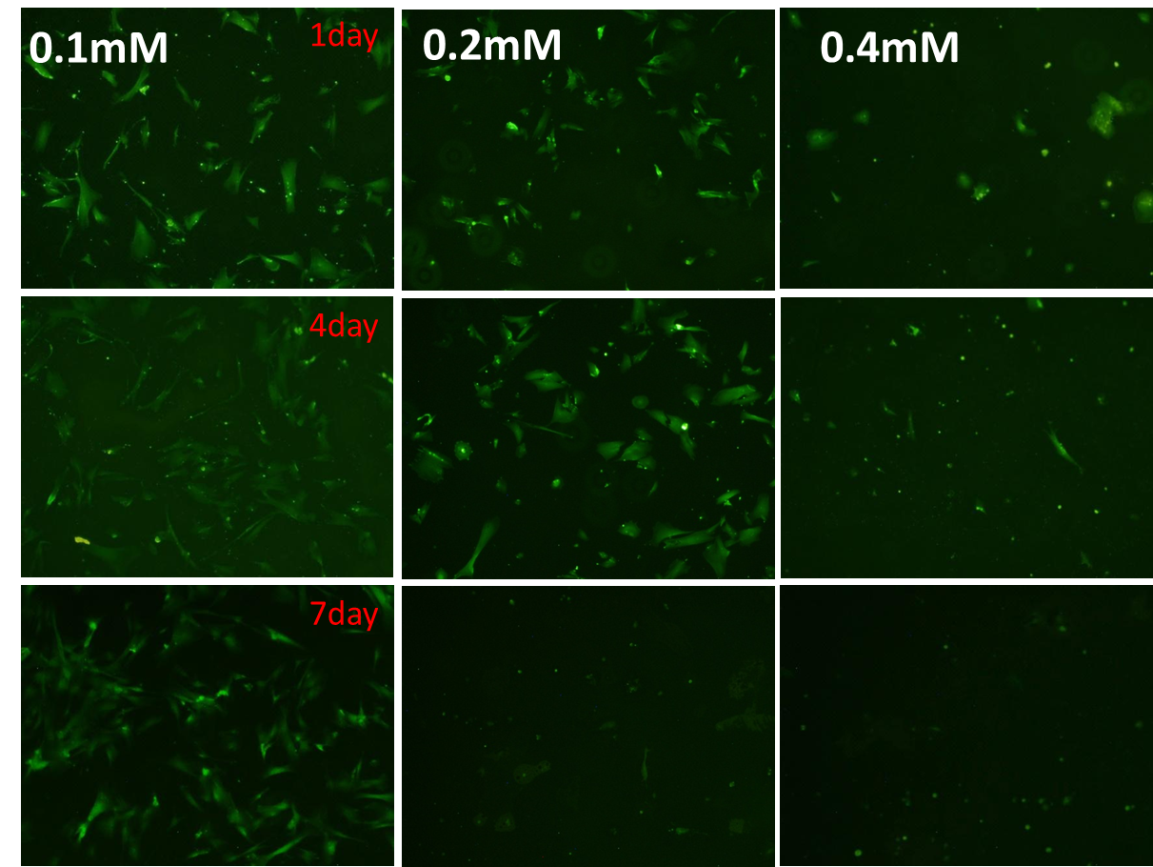


Figure S4. Smooth muscle cells cultured on plates incorporated with different EGCG concentrations.
